# Cervical Cancer and Its Association With Pregnancy

**DOI:** 10.7759/cureus.62144

**Published:** 2024-06-11

**Authors:** Swarali G Datir, Arpita Jaiswal

**Affiliations:** 1 Obstetrics and Gynecology, Jawaharlal Nehru Medical College, Datta Meghe Institute of Higher Education and Research, Wardha, IND

**Keywords:** locally advanced cervical cancer, malignant tumor, hpv, pregnancy, cervical cancer

## Abstract

Cancer is a disease in which abnormal cells divide uncontrollably, destroying tissues. A malignant tumor arises from cells in the cervix, the lower portion of the uterus (womb) that links the uterus to the vagina (birth canal), and is known as cervical cancer. One of the most significant global community health problems is cancer, which sees a daily increase in the number of sufferers. Therefore, it is crucial to expand our understanding of the molecular pathophysiology of cervical cancer and to suggest new therapeutic goals as well as new techniques for early detection of the illness. Since early diagnosis of pathologies can dramatically increase a patient's chance of survival, prognosis, and recurrence.

This article aims to educate readers about some essential concepts surrounding cervical cancer, including the various types of cervical cancer, the stages of cancer, as well as their etiology, epidemiology, pathogenesis, management, and treatment, and its relationship with pregnancy. All of these concepts are essential for any individual studying medicine or working in the medical industry to understand. We intend to summarize the information that is currently available and the recommended courses of action for treating cervical cancer and its association with pregnancy in this review. Research priorities and controversies are also noted.

## Introduction and background

The most common gynecological cancer in poor nations is cervical cancer, as it ranks third in the world in terms of cancer incidence [1]. There are two forms of cervical cancer. These include cervical squamous cell carcinomas (CSCCs), which are produced from squamous cells, and cervical adenoma, which develops from the glandular cells of the cervix [2].

Initially, the stages of cervical carcinogenesis were based on pathomorphological changes, which were later supported by research using genome-wide screening for molecular problems at various stages of cervical cancer development [3]. The staging of cervical cancer in 2018 as stated in Table 1 has recently undergone modification by the International Federation of Gynecology and Obstetrics (FIGO) [4].

**Table 1:** Stages of cervical cancer Source [4]

Stages	Description
Stage 0	Carcinoma in situ, CIN
Stage I	Invasive carcinoma confined to the cervix
Stage IA	Diagnosed only by microscopy
IA1	Microinvasive carcinoma with stromal invasion <3 mm depth, <7 mm width
IA2	Microinvasive carcinoma <5 mm depth, <7 mm width
Stage IB	Clinically visible or microscopic lesion >IA2
IB1	Clinical lesion <2 cm
IB2	Clinical lesion >2 cm and <4 cm
IB3	Clinical lesion >4 cm
Stage II	Extension beyond the cervix but not to the sidewall
IIA	Involvement of the upper two-thirds of the vagina
IIB	Parametrial involvement
Stage III	Extension to the pelvic wall and/or lower third of the vagina; hydronephrosis
IIIA	Involvement of the lower third of the vagina
IIIB	Pelvic sidewall involvement; hydronephrosis
Stage IV	Extension beyond the true pelvis or involving the bladder or rectum
IVA	Involvement of bladder or rectal mucosa
IVB	Spread outside the true pelvis or metastasis to distant organs

The most frequent malignant tumor of the female reproductive system is cervical cancer. The lives and health of women are significantly threatened by the high recurrence rate for cervical cancer patients [5]. Pregnancy termination during the first two trimesters or delaying therapy until fetal development in the third trimester, followed by postpartum conventional treatment, were the traditional approaches of treating cervical cancer. Over 500,000 women globally were impacted with cervical cancer in 2018; out of which, 300 000 people died from the disease; still, it is preventable and potentially curable. Consequently, the World Health Organization (WHO) has adopted a strategy to end cervical cancer as a public health problem, which includes Human papillomavirus (HPV) vaccination, screening, and appropriate management of preneoplastic lesions and invasive cancer. In the past 10 years, pregnancy preservation and treatment during pregnancy have become more widespread [6,7].

## Review

Etiology and risk factors

The link between high-risk genital HPV strains and CSCCs has been established by molecular and epidemiological research. The most widespread condition associated with HPV is by far cervical cancer. HPV infections in the genital tract that are persistent and high-risk account for 99.7% of occurrences of cervical cancer [8].

To examine the link between passive smoking and cervical cancer, a meta-analysis takes place which takes both factors into account. According to the findings of this meta-analysis, passive smoking advances the probability of developing cervical cancer incidentally [9].

The cervical cancer risk prediction study has not yet been reported. To offer a scientific foundation for the prevention and treatment of cervical cancer recurrence, we intend to build a risk prediction model for cervical cancer reappearance based on the factors that influence recurrence [5].

Medical and demographic factor says cervical cancer risk increases by 14 times after the age of 40 years. Organizational and medical factor says untimely medical examinations increase the chances of cervical cancer by 4-5 times. Behavioral factor includes the information that more than four sexual partners were major behavioral factors [10]. Treatment techniques, clinical stages, patient age, age of menarche, and miscarriages are all independent factors impacting cervical cancer recurrence [5]. Cervical infection with HPV is the chief risk factor for the development of cervical cancer; it is not genetically transmitted, and diet has no impact on avoiding cervical cancer [11,12].

Many of the respondents in the current study were aware of several important risk factors for cervical cancer. Even though over 70% were aware of the elevated risk associated with unprotected sexual practice or sexually transmitted diseases (STDs), only 61% appropriately recognized multiple sexual allies as a risk factor. Fewer than half of our sample knew that smoking increased risk, and even rarer knew that numerous pregnancies and prolonged use of birth control were risk factors for cervical cancer. African Americans had the highest knowledge ratings among the three racial/ethnic groups, even though multivariate evaluates that African Americans remained considerably more likely to cite a lack of information due to barriers to screening [13].

The underestimation of risk was a consistent finding in our study, as were women's lack of knowledge of cervical cancer [14]. Major developments in the understanding of the genesis of viral diseases and the risk factors for cervical cancer have given preventive medicine access to some very exciting new tools. To attain the final aim of eliminating cervical cancer, HPV vaccines and an increasing number of HPV-based screening tests must be implemented diligently and on a broad scale [15].

Prevention

The course of cervical cancer naturally presents special chances for disease prevention. Receiving an HPV vaccine to prevent HPV infection might decrease your chance of developing cervical cancer and further malignancies associated with HPV. Then Papanicolaou (Pap) tests can recognize cervix precancerous abnormalities among women who have no symptoms, permitting intensive care or treatment to avoid cervical cancer, hence advised to get it done routinely.

Additionally, screening can identify cervical cancer in its earliest stages, when there is a high chances of successful treatment. Most medical associations advise initiation of routine Pap tests at 21 years of age and having them redone every few years. The two main strategies of HPV prevention at the moment are risk reduction and vaccinations to prevent HPV infection.

Having safe sexual behavior decreases the risk of developing cervical cancer by practicing steps to avoid STDs, for example, using a latex condom each time you have sex or spermicides and limiting the figure of partners you have. After that, smoking has a very adverse impact. Hence, don't initiate smoking if you don't by now. If you presently smoke, discuss quitting methods with your doctor [8,16].

We recommend passing legislation restricting smoking in indoor areas to safeguard women, as has been done in several other nations like China and America. Given the prevalence of smoking in many nations and the rising number of cervical cancer patients globally, quitting smoking should be seen as an essential public health measure to stop and manage the global cervical carcinoma epidemic. Further initiatives to enforce the terms of the WHO Framework Convention on Tobacco Control have been adopted to limit smokers' current tobacco usage. A smoke-free policy may provide protections for those who have never smoked and may boost the number of people who successfully stop [9].

Pathogenesis

Cervical cancer is generally understood to be a multistage process involving uncontrolled cell growth that has undergone malignant transformation. Phenotypically, it begins with early tissue changes called hyperplasia, advances to dysplasia, carcinoma in situ, and finally becomes an invasive cancer that can metastasize and spread to distant tissues via the lymphatic and blood systems [17].

Through the limitation of HPV and the accumulation of transmutations in cancer driver genes, APOBEC is the fundamental protein particle in the pathogenesis of cervical cancer. Additionally, it has been noted that the distribution of the APOBEC3A/3B deletion polymorphism in the Southeast Asian population influences cancer incidence. However, it defies logic because there is less chance of a greater mutation rate in the case of the total deletion of APOBEC3B, a potent nuclear DNA mutator enzyme. Additionally, the relationship between the APOBEC3A/3B deletion polymorphism and cervical squamous cell cancer has not yet been thoroughly studied. To fully understand its relationship to cancer risk, viral restriction, and mutagenesis, this deletion polymorphism will need to be studied in various populations in the future [2].

Clinical manifestation and management

Early-stage cervical cancer typically has no symptoms or indicators. The following are symptoms and signs of more advanced cervical cancer: vaginal bleeding following sex, in between cycles, or after menopause; heavy, bloody, and/or smelly vaginal discharge that is watery and bloody; and discomfort during sex or pain in the pelvis [16].

Investigation

Physical examination: Evaluation of the pelvic lymph nodes. The utmost accurate method for determining the nodal status of the lymph nodes continues to be histopathologic evaluation. Among the most important prognostic indicators for patients with cervical cancer is the lymph node status. The lymph node status can direct management, particularly in cervical cancer that is in its early stages.

Magnetic resonance imaging (MRI): MRI is the standard diagnostic technique that is practiced to evaluate lymph node infiltration, vaginal and parametrial invasion, stromal invasion, and tumor size in three dimensions.

Ultrasound: Sonographic evaluation of local disease extension has recently been demonstrated to be practical for preoperative staging. Transrectal or transvaginal ultrasound has been proven in certain prospective trials to be comparable to the MRI's diagnostic efficacy [7].

Pap testing has served as the effective backbone of initiatives to prevent cervical cancer for many years [18].

Treatment

The following is a description of the typical cervical cancer therapies. Treatment for symptoms and side effects (a vital component of cancer care) may also be included in your care plan. Types of treatment include radiation therapy, surgical procedures, and immunotherapy [19].

Radiation therapy: Radiation therapy is crucial in the management of locally advanced cervical cancer. Radiotherapy for cervical cancer abolishes ovarian functions and fertility due to the administration of high dedication to the uterus. Within the last 20 years, there has been an outstanding improvement in radiation oncology techniques. Particularly, the application of image-guided intensity-modulated radiotherapy (IG-IMRT) and three-dimensional image-guided adaptive brachytherapy (3D-IGABT).

IG-IMRT: In comparison to traditional two-dimensional (2D) or 3D-conformal radiotherapy (3DCRT) techniques, IMRT allows for the delivery of a highly conformal dose distribution while minimizing the dose to nearby organs at risk (OARs). This leads to an improved toxicity profile, with no patient experiencing grade 3 toxicity and a decrease in grade 2 acute gastrointestinal toxicity in the IMRT group compared to the conventional whole pelvis RT group.

3D-IGABT: One kind of radiation therapy (RT) called brachytherapy (BT) uses tiny, sealed radioactive sources to deliver a therapeutic dose inside or close to a tumor volume. BT for laparoscopic approach to cervical cancer (LACC) was previously administered via a 2D method. 2D-BT provides a known dose to point A bilaterally, producing a pear-shaped distribution, but these cannot be easily determined from an X-ray. Hence, it does not account for patient-specific characteristics such as tumor size, anatomy, or doses to OARs. However, the administration of BT has changed to a volume-based method, accounting for changes in tumor size and position throughout the course of treatment, with the introduction of CT and MRI imaging during the process.

Hence, IG-IMRT and 3D-IGABT are currently observed as the gold standard in the majority of countries and have significantly enhanced treatment results and toxicity profiles for patients with locally advanced cervical cancer [20].

Surgical procedure: In the treatment of cervical cancer in its early stages, surgery is crucial. The secret to effective results is cautious patient selection. The standard method is still a conventional type III open radical hysterectomy with bilateral pelvic lymph node dissection. Recent research strongly suggests that open surgery should be used instead of minimally invasive surgery for radical hysterectomy. Highly selected patients may be able to preserve their fertility. In a young patient undergoing a radical hysterectomy, the ovaries may be preserved. Less invasive surgical procedures should not be recommended outside of a study for patients suffering from early-stage cervical cancer who have favorable prognostic indicators, as this is still under investigation [21].

Immunotherapy: Although the overall response rate for several of these methods is low, the sustained response for some patients seems to be long-lasting. These medicines are probably going to be the primary treatments for first-line metastatic illness or even as sensitizing agents in combination with initial therapy to avoid recurrence given the limited alternatives for patients with recurrent cervical cancer. It will be necessary to balance the possible toxicity of these medications with the ideal combination and regimen [22]. Overall, as we learn more about the biology of the disease, particularly genetics, and immunology, the paradigm for systemic treatment of cervical cancer is steadily shifting. Concurrent chemoradiotherapy (CCRT) continues to be the standard of care for locally advanced cervical cancers, although neoadjuvant chemotherapy (NACT) followed by local therapy has been suggested to be helpful but needs further research to be confirmed. Chemotherapy regimens based on cisplatin are still the gold standard for treating metastatic illness, and bevacizumab addition has been demonstrated to increase survival. Pembrolizumab is an immunotherapy drug that has the potential to treat advanced disease [23].

Association of cervical cancer with pregnancy

In expectant women, there is always a connection between the tumor diameter and clinical stages of malignancy with the clinical symptoms of cervical cancer. Early cervical cancer during the expectancy period is typically without significant clinical symptoms, although a small number of symptomatic patients generally display stenchy vaginal discharge, purulent or bloody discharges, and irregular vaginal bleeding. Chronic anemia carried on by long-term irregular vaginal bleeding or pain from tumors are the main symptoms of pregnancy with late cervical cancers. The previous symptoms are often confused with those of other ailments during pregnancy or puerperium since such females are either pregnant or postpartum. Therefore, one should be extremely cautious while treating pregnant patients and postpartum patients who have vaginal bleeding, and if necessary, a gynecological examination and cervical exfoliation cytology screening should be done [24].

Decisions about the diagnosis and treatment of malignancies in pregnancy must carefully consider pregnancy physiology, dynamic anatomy, and fetal considerations when it comes to management. As the corpus of research on cancer during pregnancy expands, doctors can find tips for achieving excellent oncologic and risk-free obstetric results [25]. There is a “three-step” model followed during screening for pregnancy with cervical cancer, and it includes cervical cytology, colposcopy, and cervical biopsy [24]. If an ultrasound expert is available, this can be a good alternative to MRI during pregnancy [7]. As we know, radical hysterectomy is the kind of decided treatment for women with early-stage disease, still, we should give thought to women's benefit by selecting adjuvant radiation to further decrease their risk of cancer reoccurrence [26]. Many cervical cancer survivors remain filled with the hope of having children in the future. Vaginal radical trachelectomy (VRT) exhibits positive reproductive outcomes in early-stage cancer without sacrificing oncological safety. Gestational surrogacy offers a viable alternate choice for achieving a biological child for persons who need to receive gonadotoxic treatment [27].

The treatment of cervical cancer in pregnant women generally follows similar guidelines as in patients who are not pregnant. The treatment of patients is started right away before 16 to 20 weeks of pregnancy. Depending on the disease's stage, the technique of treatment might either be surgery or chemotherapy. Radiotherapy is not involved as conceptus frequently spontaneously aborts as a result of radiation. Radiation therapy takes center stage in the course of treatment after the tumor reaches an advanced stage. The result of this therapy is maximum 40% and minimum less than 20% for stages IIb, III, and IV, respectively. In order to manage their systemic illness, patients with distant metastases (stage IVb) also need chemotherapy. There has been continuous research to increase the survival rate in cases with advanced-stage cervical neoplasia. From the late second trimester onward, certain instances can be treated with surgery and chemotherapy, while the pregnancy is still viable. Delaying final treatment is an option for stages IA2, IB1, and IB2 when the diagnosis is established after 20 weeks, and it has not been demonstrated to have any detrimental effects on the prognosis when compared to nonpregnant patients. The interests of both the mother and the fetus must be balanced while choosing the right delivery time. Delivery by traditional cesarean section with radical hysterectomy at the same time is performed no later than 34 weeks of gestation when delivered at a tertiary facility with suitable newborn care. The effect of therapy delaying survival in more severe diseases is unknown. When a treatment delay is intended, NACT may be given to women with locally advanced cervical cancer to stop the disease from progressing [28]. Even if the method of delivery is strongly debated, delivery should be carried out as soon as the fetus's pulmonary maturity is apparent. Due to the possibility of disease recurrence at the episiotomy site and the increased risk of hemorrhage, obstructed labor, and infection associated with delivery through a cervix with advanced cervical cancer, the majority of specialists advise cesarean delivery [29].

Fetal growth and neonatal development were not visibly impacted by abdominal radical trachelectomy during pregnancy (ART-DP) for pregnancy-associated cervical cancer. All fetuses were capable of escaping exposure to anticancer drugs after ART-DP. We need to keep looking into perinatal results and kids' growth and development following ART-DP to establish its safety and efficacy for pregnancy-associated cervical cancer [30]. Anticancer medications concentrate on breast milk and can have an impact on the newborn. Therefore, breastfeeding is not advised for women undergoing chemotherapy [25].

## Conclusions

The approaches discussed here, along with the potential for a new generation of treatments combined with CCRT, should improve access to care worldwide and help patients with locally advanced cervical cancer achieve their objectives, i.e., it has improved survival and reduced the risk of recurrence. The trials performed have shown some promise, and we look forward to the results.

Pregnancy-related cervical cancer symptoms are infrequent, challenging to recognize, commonly misdiagnosed as diseases associated with pregnancy, and easily masked by the presence of a pregnancy. Cervical cancer screening is done in three steps to prevent disregarding these instances. After the case is diagnosed, we can combine the clinical stages of the patients, lymph node status, histological types of tumors, gestational weeks, imaging data, and the patients' and their families' willingness to become pregnant to weigh the advantages and disadvantages and develop a personalized treatment plan. In cases of complicated cervical cancer pregnancies, it is the best option. There is presently no standardized approach to treating cervical cancer linked to pregnancy.

In the past 10 years, research for the treatment of cervical cancer has advanced. The emphasis is still on immunotherapy therapies, fertility preservation, and prevention. To continue tracking obstetric outcomes and potential immune-related toxicities in this patient cohort, more studies will be required.
